# The Development of a Novel Questionnaire Approach to the Investigation of Horse Training, Management, and Behaviour

**DOI:** 10.3390/ani10111960

**Published:** 2020-10-24

**Authors:** Kate Fenner, Katherine Dashper, James Serpell, Andrew McLean, Cristina Wilkins, Mary Klinck, Bethany Wilson, Paul McGreevy

**Affiliations:** 1 Sydney School of Veterinary Science, University of Sydney, Camperdown NSW 2006, Australia; bethany.wilson@sydney.edu.au (B.W.); paul.mcgreevy@sydney.edu.au (P.M.); 2 School of Events, Tourism and Hospitality Management, Leeds Beckett University, Leeds LS1 3HE, UK; K.Dashper@leedsbeckett.ac.uk; 3 School of Veterinary Medicine, University of Pennsylvania, Philadelphia, PA 19104, USA; serpell@vet.upenn.edu; 4 Equitation Science International, 3 Wonderland Ave, Tuerong, Victoria 3915, Australia; andrewmclean@esi-education.com; 5 Saddletops Pty Ltd., Gatton, Queensland 4343, Australia; cristinaluz@horsesandpeople.com.au; 6 Dre Mary Klinck, m.v., consultante en comportement, Sainte-Anne-de-Bellevue, QC H9X0A6, Canada; maryklinck@hotmail.com

**Keywords:** horse welfare, rider safety, behavioural evaluation, citizen science, ethical equitation, the domestic equine triad

## Abstract

**Simple Summary:**

The way horses are trained and managed influences their behaviour. The Equine Behaviour Assessment and Research Questionnaire (E-BARQ) was developed to gather information on the training, management, and behaviour of domestic horses. An international panel was established to assist with the questionnaire development and the pilot questionnaire collected data on 1320 horses. Statistical analysis revealed the E-BARQ to be a suitable representation of relevant features of horse training and management and the objective reporting of horse behaviour.

**Abstract:**

The Equine Behaviour Assessment and Research Questionnaire (E-BARQ) is a questionnaire instrument developed to obtain quantitative data on the domestic equine triad of training, management, and behaviour of horses. The E-BARQ was developed to identify how changes in training and management impact behaviour over time, to define normal behaviour in horses, and to discover how to improve rider safety and horse welfare, leading to ethical equitation. During the development of the E-BARQ, we also investigated how best to motivate stakeholders to engage with this citizen science project. The pilot version of the E-BARQ collected qualitative data on respondents’ experience of the questionnaire. The pilot questionnaire was developed with the assistance of an international panel (with professional expertise in horse training, equitation science, veterinary science, equestrian coaching, welfare, animal behaviour, and elite-level riding), and was used to collect data on 1320 horses from approximately 1194 owner/caregiver respondents, with an option for respondents to provide free-text feedback. A Rotated Principal Component Analysis of the 218 behavioural, management, and training questionnaire items extracted a total of 65 rotated components. Thirty-six of the 65 rotated components demonstrated high internal reliability. Of the 218 questionnaire items, 43 items failed to reach the Rotated Principal Component Analysis criteria and were not included in the final version of the E-BARQ. Survey items that failed the Rotated Principal Component Analysis inclusion criteria were discarded if found to have a less than 85% response rate, or a variance of less than 1.3. Of those that survived the Rotated Principal Component Analysis, items were further assigned to horse temperament (17 rotated components), equitation (11 rotated components), and management and equipment (8 rotated components) groups. The feedback from respondents indicated the need for further items to be added to the questionnaire, resulting in a total of 214 items for the final E-BARQ survey. Many of these items were further grouped into question matrices, and the demographic items for horse and handler included, giving a final total of 97 questions on the E-BARQ questionnaire. These results provided content validity, showing that the questionnaire items were an acceptable representation of the entire horse training, management, and behavioural domain for the development of the final E-BARQ questionnaire.

## 1. Introduction

Over centuries, the role of the horse in society has changed dramatically, so too have the ways we train and manage domestic horses. In the developed world, horses have moved from being utility-based working animals to sport, leisure, and companion animals, spending less of their lives as beasts of burden and more time in close contact and interaction with humans in the leisure industry. As many traditional training and management methods are now outdated, and often inappropriate, modern practices have developed in a somewhat haphazard manner, at times incorporating antiquated folklore, obsolete techniques, and potentially unethical practices [1]. This highlights the need for evidence-based practice that prioritises horse welfare [1] and ethical equitation [2]. The ways we train and manage horses will always have a direct impact on their behaviour, which in turn affects both rider safety and horse welfare [3].

Despite their altered position in society, and increasing roles in companionship and leisure, the welfare of horses, our understanding of their behaviour, and optimal training and management practices, seem to lag behind that of their canine counterparts [1,4]. Horses have the unique position of being the only animal used in Olympic sports, yet it could be argued that the rules protecting their welfare [5] in such sports are insufficient, inadequately defined, and patchily enforced [4,6]. Even at the elite levels of sport and competition, horses may be subjected to questionable practices, including the use of cervical hyperflexion (also known as Rolkur) [7], restrictive nosebands [8], and the over-application of artificial aids [9] that are proving difficult to justify and that, in many jurisdictions, would bring about prosecution if imposed on other species, such as dogs [10].

Most equids in the developed world today are companion and leisure riding horses, a markedly different horse-human dyad from the days of their military and agricultural roles [11]. However, problems may arise when leisure riders seek to emulate those professionals’ training and management regimes in addition to the tack and equipment used [12]. For example, despite evidence that horses are sensitive to social deprivation [13], many amateur dressage riders will confine their horses to contain energy and capitalise on the perceived benefits of post-inhibitory rebound in locomotory behaviour when taken out of the stable [14,15]. The absence of a large-scale database describing current practice in training and management, and any objective means of benchmarking *normal* behaviour in horses [16], or indeed, the value of collecting such data, has led to considerable confusion among leisure horse owners and riders.

Horses with undesirable or dangerous behaviours are increasingly likely to be subjected to positive punishment techniques [17], or be sold, rehomed, or euthanised [18]. Thus, a horse’s behaviour directly affects its welfare. There is evidence that the sale price of horses reflects the risk their behaviour represents [19,20,21]. Training and management practices influence behaviour [22], and with evidence that over 91% of leisure riders in the UK experience problem behaviours when riding [23], and with horse-related activities leading to 1600 emergency presentations in Victoria, Australia, in 2015–2016 [24], it is important to collect large scale data on equine behavioural problems to protect rider and handler safety, and horse welfare.

In 2011, Hawson et al. [19] investigated whether rider safety was valued in the Australian pony market and found that safety descriptors did not contribute to the pricing of ponies in Australia but warnings did. Some seven years later, O’Connor et al. [24] reported that emergency department presentations, for horse riding injuries in Victoria, Australia, had increased by 28.8% and hospital admissions by 47.6% from 2002 to 2016 and suggested the need to refocus on injury prevention. It has been stated that riders can expect to have one injury per 100 h of leisure riding and one injury per hour of cross-country eventing [25]. This is in contrast to the expected rate of injury for motor-cyclists of one injury per 7000 h [25]. Injury prevention would greatly benefit from a deeper understanding of how our training and management practices influence behaviour. This understanding, coupled with educational campaigns to disseminate the information, should improve rider safety.

There is a paucity of literature on both the rates of, and reasons for, so-called “wastage” in the equestrian industry. In 2002–2003, it was reported that almost 40% of Thoroughbred and Standardbred racing horses left racing in Australia, with their trainers citing ‘poor performance’ as the main reason and ‘unsuitable behaviour’ accounting for just 6.4% of that wastage [18]. In the leisure riding industry, which is not a performance-based sector, behaviour is thought to be the principal reason horses are sold, re-homed, or euthanised [26]. However, the true wastage rates and reasons remain unknown, as longitudinal data on these animals is not available, or easily obtained.

The current project is modelled on the Canine Behavioral Assessment and Research Questionnaire (C-BARQ) [27], a citizen science project that has contributed substantially to our understanding of dog behaviour. The C-BARQ is a standardised and validated behavioural assessment tool that has been used in more than 100 published research studies. The C-BARQ database at the University of Pennsylvania (www.cbarq.org) currently contains behavioural evaluations of over 60 thousand companion dogs and a similar number of working assistance dogs, and represents a unique resource for canine scientists and other dog-oriented groups. Research findings based on the C-BARQ have challenged traditional and long-held beliefs about canine behaviour, including revelations regarding the behavioural associations of body size and head shape [28], the influence of de-sexing [29,30], and the genetic basis of breed differences in canine temperament [31,32]. The C-BARQ was originally developed as a proxy measure of variation in canine behaviour and temperament. Its equine equivalent, the Equine Behavioural Assessment & Research Questionnaire, seeks to fulfill a similar role but, because horses are ridden and driven by humans, the E-BARQ also aims to incorporate aspects of horse-rider/horse-handler interaction in addition to items relating to horse behaviour and temperament.

E-BARQ has the potential to fill a number of current knowledge gaps. First, to determine how early training and management practices affect later behaviour, it is necessary to collect longitudinal data. Second, interactions with horses, particularly when we ride or drive them, can be high-risk activities, and only by understanding what normal behaviour is, can we predict when a horse might behave dangerously. Longitudinal data will reveal the early predictors of behavioural differences that, if left unaddressed, may manifest as dangerous behaviours in later life. They will also reveal how horses of different breeds may vary during maturation. E-BARQ also asks respondents to supply photographs of their horses’ heads, facilitating studies that relate head-shape to behaviour (as have been published for dogs [28,33]).

This article describes how the E-BARQ survey was developed and enhanced using Rotated Principal Component Analysis and the incorporation of stakeholder feedback, to produce the final version of the questionnaire.

## 2. Materials and Methods

### 2.1. The Questionnaire

To ensure initial content validity, the E-BARQ pilot questionnaire was developed with the assistance of an international panel of nine representatives from the fields of veterinary science, horse training, horse welfare, elite level competition, equestrian coaching, equitation scientists, and equine behaviourists. The questionnaire contained closed-ended items, including 41 demographic questions about both horse and owner/caregiver, and then branched into two sections with 268 ridden horse items (if the horse had been ridden or driven in the last six months), and 218 non-ridden horse items (the horse had not been ridden or driven in the last six months), respectively (see Supplementary File S1; all participants responded to the same 218 non-ridden items while ridden-horse respondents had to respond to an additional 50 items investigating behaviours under saddle). An open-ended question for respondents to offer feedback was provided at the conclusion of the questionnaire.

E-BARQ respondents were resident in 20 different countries, including Australia (*n* = 385), the United Kingdom (*n* = 294), the United States (*n* = 202), and Canada (*n* = 114). Respondents self-classified into rider experience levels of ‘non-rider’ (*n* = 3), ‘beginner rider’ (*n* = 27), ‘novice rider’ (*n* = 145), ‘intermediate rider’ (*n* = 654), ‘advanced rider’ (*n* = 459) and ‘elite rider’ (*n* = 18), with 13 missing values. Over 90% of respondents were 25 years of age or older. Horses were described as being involved in thirty-four riding disciplines, including dressage (*n* = 288), trail riding/hacking (*n* = 285) and pleasure riding (*n* = 160).

The questionnaire was built using REDCap electronic data capture tools hosted at the University of Sydney and accessed via a URL link [34]. Respondents could complete the survey in a single session or ‘save and return’ as necessary. Because E-BARQ was designed as a longitudinal study and a repeatable instrument, it was important that respondents only reported on behaviours the horse had exhibited in the preceding six months. The relevant period, together with the horse’s name, appeared in each question to remind the user of the timeframe and which horse, if they had more than one, they were reporting on.

The E-BARQ pilot questionnaire was distributed to an audience of horse enthusiasts via Facebook posts and the email lists of *Horses and People Magazine* (https://horsesandpeople.com.au/), *Equitation Science International* (https://www.esi-education.com/) and *Kandoo Equine* (https://www.kandooequine.com/). Members of the international panel also assisted with the distribution within their own networks. The questionnaire URL link was removed when 1320 completed responses had been obtained for the current analysis.

### 2.2. Rotated Principal Component Analysis

The Rotated Principal Component Analysis was performed using the psych package (Revelle, W. (2019) psych: Procedures for Personality and Psychological Research, Northwestern University, Evanston, IL, USA, https://CRAN.R-project.org/package=psych version 1.9.12.) of R statistical and computing software (R Core Team, 2020. R: A language and environment for statistical computing. R Foundation for Statistical Computing, Vienna, Austria. URL https://www.R-project.org/.) employing varimax rotation. The number of components to extract and rotate were selected by the parallel analysis algorithm.

Rotated Principal Component Analysis is a method used to classify variability among correlated variables and thereby find independent latent, not directly measured, variables. The analysis was undertaken to reduce the question item numbers and thus streamline the survey. Analysis was conducted ‘blind’ with respect to the proposed rotated component classifications that had been envisaged apart from being performed separately on each sub-questionnaire.

Due to the complexity of the human-equine relationship, and the increased risk of spurious rotated components when modelling such a complex construct, for the purposes of construct validation, the E-BARQ Questionnaire was divided into three subsections: (1) Management (E-BARQ-m), (2) Equitation (E-BARQ-e), and (3) Temperament (E-BARQ-t).

Identification of underlying components was undertaken using 53 survey items for E-BARQ-m, 53 items for E-BARQ-e, and 107 items for E-BARQ-t. Varimax rotations were applied to the principal components to simplify interpretation of the underlying structure. A scree plot of a parallel analysis, using R’s fa.parallel function, was then examined to investigate the number of rotated components in the data matrix.

Based on Bartlett’s Test of Sphericity, each sub-questionnaire was considered suitable for the application of a data reduction technique to identify underlying components. (Ridden Management sub questionnaire (E-BARQ-m), χ^2^ = 12,745.014, df = 1378, *p* < 0.001; Equitation Ridden sub questionnaire (E-BARQ-e) χ^2^ = 21,884.714, df = 1653, *p* < 0.001; Temperament Ridden sub questionnaire (E-BARQ-t) χ^2^ = 48,978.677, df = 5671, *p* < 0.001). The Kaiser-Meyer-Olkin test was used to evaluate sampling adequacy for data reduction analysis and was deemed acceptable with overall Measures of Sampling Adequacy (MSA) of E-BARQ-m = 0.71, E-BARQ-e = 0.80 and E-BARQ-t = 0.77.

Questions were considered for exclusion if they failed to load strongly (>0.5) onto one of the extracted Varimax components, and if they failed to reach the threshold of an 85% response rate or had a variance of less than 1.3, and were not of prima facie interest (see Supplementary File S2).

### 2.3. Qualitative Data

Respondents were invited to leave feedback in an open textbox. This was designed to capture any additional items that the respondents suggested should be in the survey, and to gather general feedback on the questionnaire.

## 3. Results

### 3.1. Identification of Underlying Components

Examination of a scree plot of a parallel analysis, using R’s fa.parallel function, investigated the number of rotated components in a data matrix, and was then used to determine the number of components to extract for rotation resulting in 8 for E-BARQ-m (see Table 1), 14 for E-BARQ-e (see Table 2), and 17 for E-BARQ-t (see Table 3). This criterion was more stringent than the so-called Kaiser rule, which excludes all rotated components with (un-rotated) eigenvalues less than 1 which would have resulted in 15 for E-BARQ-m, 17 for E-BARQ-e, and 33 for E-BARQ-t. The extracted components accounted for 52% of total variance E-BARQ-m, 58% of total variance for E-BARQ-e, and 62% of the total variance for E-BARQ-t. Because of their considered importance, some items were retained despite not meeting the inclusion criteria and are listed as non-loading and miscellaneous items in Table 1, Table 2 and Table 3 (items that were cut from the survey can be found in Supplementary File S2).

#### 3.1.1. E-BARQ-m Components and Retained Questions

The eight maintenance-and-gear rotated components included the type of saddle, bit and noseband used, pre-ride exercise requirements, maintenance procedures undertaken, and the time the owner spent with the horse (see Table 1).

#### 3.1.2. E-BARQ-e Components and Retained Questions

The 11 Equitation rotated components are those exhibited when being ridden or driven (see Table 2). They included items such as bridle-related conflict, responsiveness under saddle, response to steering, deceleration, rearing, bucking, and the horse’s response to verbal cues. These items were grouped to represent the horse’s behaviour both under saddle and when being worked on the ground.

#### 3.1.3. E-BARQ-t Components and Retained Questions

The 17 Temperament rotated components pertained to behaviours that horses exhibited on the ground when worked or when not interacting with a handler (see Table 3). They included items such as shyness from man-made objects, secure in familiar environments, reactive to non-equine animals, compliant with unfamiliar human care interventions, defensive to other equines and insecure in challenging environments. These items were grouped to represent the horses’ general behaviour.

### 3.2. Qualitative Feedback

The final item on the questionnaire, an open textbox option, provided qualitative feedback on the survey that alerted developers to anything that was missing from the survey, as well as question items seen as particularly important to participants. Most respondents chose to tell us more about their horses and any associated issues. Text responses were grouped into four categories: additional details about the horse (21.9% left positive comments on horse temperament, and 11.1% stated how much the respondent loves the named horse); management (20% provided extra information about management practices), training (23% provided detail on training methods) and veterinary concerns (14.2%); issues with the horse (divided into behavioural issues, mentioned by 44.2%, and performance issues, mentioned by 6.4%); and comments about the survey itself (praising the survey—15.5%; identifying specific issues—13.3%; and making suggestions for further questions/options—8.6%). The most common category was ‘issues with the horse’, with responses focusing predominantly on behavioural issues, specifically those related to perceived nervousness (13.6%) and aggression (7.5%) in the horse. The prevalence of such responses reinforces the need for E-BARQ to help guide handlers/riders to develop training and management practices aimed to minimise the development and severity of behavioural issues, which are problematic in terms of rider/handler safety and horse welfare. Open text responses were optional, but 360 respondents to the pilot survey (36.8%) chose to leave an answer, illustrating engagement with the idea and development of E-BARQ.

## 4. Discussion

The Rotated Principal Component Analysis was an essential component of building the E-BARQ questionnaire. The analysis allowed the research group to streamline the survey. Daily training and management regimes that fail to reflect a horses’ ethology greatly affect behaviour, rider and handler safety, and horse welfare. A vast gap in our knowledge exists in this very relationship and while science has made inroads in the areas of equitation, relatively little is known about the long-term relationship of these elements. Questionnaire instruments such as the E-BARQ provide an opportunity to close some of these gaps in current knowledge. The Rotated Principal Component Analysis described here identified 53 rotated components providing construct validity for the final version of the E-BARQ questionnaire.

Of the 218 items included in the Rotated Principal Component Analysis, 128 met the inclusion criteria, 43 failed and a further 47 failed to meet the criteria but were retained for their considered importance and interest to the ongoing research of the domestic equine triad. For example, some non-loading and miscellaneous items in M_1 (see Table 1) describe the amount of time respondents spend with their horses and whether they train using food rewards (an increasingly utilised form of positive reinforcement in horse training). Both items were considered of importance to the E-BARQ database and thus retained despite, not meeting the criteria. Other items in the management survey that were also included despite failing the RPCA pertained to the type of tack and equipment riders used. This can vary among disciplines and geographically and is likely to affect horse behaviour.

We found that several items that were considered important failed to load amongst the equitation components. These included whether the horse bolted, lacked forward movement, refused jumps, pulled back when tied, rushed through narrow spaces, and reared when loading. Each of these items can have an immediate and detrimental impact on handler or rider safety and horse welfare and, as such, they were included in the final E-BARQ version. The equitation components contain what may be considered the only subjective questions in the E-BARQ questionnaire. These pertain to how the owner or rider considers their horse learns in response to various types of reinforcement and punishment. While the pressure-release and correction (E_10) items loaded, whether the horse was thought to learn quickly using food rewards did not, but was included in the final version because of its considered importance to future researchers. Owners’ perception of how quickly a horse learns may well be reflected in the treatment it receives, and thus impact behaviour over time.

Several temperament components were retained despite low Cronbach Alpha scores because of their considered importance for rider safety and horse welfare. Other items in this group were combined to reduce the final question numbers. For example, respondents had been asked how their horses reacted to novel objects both when ridden or handled and when not handled. This matrix now inquires whether horses strongly avoid such objects at any time. Horses ‘spooking’ is known to be a major cause of injury to riders, with an estimated 26.69% or all horse-related injuries arising from this behaviour [35]. Windsucking and crib-biting were combined, and a definition provided as it may have been confusing some respondents. Items focusing on defensive and aggressive behaviours, while not consistently scoring high Cronbach Alphas, were included, again, for their importance in improving rider safety and horse welfare.

Feedback from the E-BARQ pilot study demonstrated how invested many horse owners are in their horse’s health, well-being, and training. As mentioned, E-BARQ is already a relatively lengthy survey to complete, yet 36.8% of respondents to the pilot survey still chose to leave additional comments, often revealing personal details about their horse. Many of the responses reported on ‘problems’ encountered with specific horses, such as behavioural or veterinary issues, but many also included praise and affection for individual horses. Among respondents there was clear interest in at least trying to do the best for their horse’s welfare and well-being, even if this was not always successful. This reinforces the need for projects such as E-BARQ, which can provide well-meaning, if sometimes ill-informed, horse owners with information, advice, and some support in developing horse management and training practices that will promote better welfare.

The final version of the E-BARQ was built on the Qualtrics [36] platform due to its enhanced user-interface and mobile accessibility. A WordPress website was built to provide users with a password-protected dashboard and access to graphs on their results, as a form of feedback, and to facilitate engagement with the project (https://www.e-barq.com/).

To investigate wastage rates and the reasons horses may change homes, a brief ‘exit’ survey was developed. This was made accessible on participants’ dashboards and could be accessed by all registered E-BARQ users. Users were required to complete the exit survey to remove the horse’s record from their profile.

The final version of the E-BARQ survey was translated into Spanish and French to improve accessibility. The initial translation was made using Google Translate, within the Qualtrics platform. This was then edited by fluent language, equine enthusiasts to maintain a true and accurate representation of the original English version of the questionnaire. Both translators found most of their editing was required with equine-specific terms pertaining to tack and equipment and horse breeds and colours. The Spanish translation, while suitable for Europe, was not suitable for Latin American speakers and a further translation is planned for this region.

Many horse owners care deeply for their horses and see them as individuals with whom they develop close relationships [37]. The E-BARQ asks for and then uses a specific horse’s name throughout, which helps keep the respondent focused on that horse when answering questions and also creates a sense of personalisation. In the pilot survey, respondents used an individual horse’s name in 38.1% of text feedback comments, possibly suggesting a positive response to the personalisation feature of the survey. This level of personalisation may encourage ongoing engagement with the E-BARQ.

The collection of data via online survey is always subject to limitations [16] and, while the E-BARQ has been developed to mitigate these as far as possible, they must be acknowledged. These authors acknowledge that this, and future E-BARQ investigations, aim to explore correlations between variables rather than causative relationships. Data collection relies on owner-reported observations and recall bias could impact the quality of these data as owners are reporting on a six-month period. Confirmation bias, where survey respondents’ experience influences their observations, may also be a limitation in some cases, despite the E-BARQ validation assessment suggesting otherwise [38]. Finally, while respondents are assured that their information will remain confidential, some may be disinclined to answer questions truthfully if they consider responses not to show them in the best light.

## 5. Conclusions

The Rotated Principal Component Analysis revealed just how complex the horse-human dyad can be, particularly when horses are ridden or driven. Given this complexity, it was necessary to divide the E-BARQ survey into three distinct parts—management, equitation, and temperament—to extract all the required rotated components. While the analysis revealed 43 items that could be removed from the questionnaire, 47 items that failed to meet the selection criteria were included because of their considered importance to rider safety and horse welfare. Each of the items that remain on the 97-question E-BARQ survey provides essential information that will help us define what normal behaviour for horses is, predict risks for unwanted or dangerous behaviours, promote ethical equitation, and protect rider safety and horse welfare.

## Figures and Tables

**Table 1:** Extracted management rotated components. SL—strongly loading items (*n*); Alpha—Cronbach Alpha of the number of strongly loaded items; Outcome: Retained—all questions were retained for the final version of the E-BARQ.

E-BARQ-m Components	SL	Alpha	Outcome
1. Rider gender, experience, and age	5	0.81	Retained
2. Tack used (e.g., rein types, crupper and standing martingale)	5	0.66	Retained
3. Groundwork	4	0.66	Retained
4. Tack used (Western and English saddles and Western curb bit)	3	0.72	Retained
5. Outings (trailer, show, trail riding)	3	0.54	Retained
6. Tack used (English curb and double bridle)	2	0.65	Retained
7. Tack used (e.g., bitless bridle, snaffle bit, treeless saddle)	5	0.67	Retained
8. Foot maintenance (professional and non-professional trimming)	2	0.57	Retained
**Non-loading and Miscellaneous Items**			
Frequency of training types, noseband and bridle types, shoeing frequency, time spent with horse, maintenance procedures, saddle and bit types	24		Retained

**Table 2:** Extracted equitation rotated components. SL—strongly loading items (*n*); Alpha—Cronbach Alpha of the number of strongly loaded items; Outcome: Retained—all questions were retained for the final version of the E-BARQ.

E-BARQ-e Components	SL	Alpha	Outcome
1. Response to rein signals (e.g., raise or toss head, pull on reins, brace neck, move faster)	5	0.8	Retained
2. Trailer loading (e.g., truck and trailer, loading speed, rushing off)	5	0.86	Retained
3. Responsiveness to leg, seat and rein (e.g., leg pressure and seat to accelerate, rein to turn)	7	0.8	Retained
4. Rearing (covering both frequency and environment)	4	0.75	Retained
5. Fail to stop or slow when signalled (with rein or lead rope signal)	4	0.78	Retained
6. Refuse to cross water (when ridden and led)	2	0.9	Retained
7. Pull back and throw head (when bridling and unbridling or haltering)	3	0.65	Retained
8. Pulling on lead and pushing handler (with food and when leading)	4	0.76	Retained
9. Artificial aids (whip and spurs)	2	0.59	Retained
10. Buck (frequency and situation)	3	0.77	Retained
11. Responsiveness to voice signals (acceleration and deceleration)	2	0.63	Retained
**Non-loading and Miscellaneous Items**			
Steering, bolt, jump refusal, seat cues, food rewards, pulling back when tied, rear when loading, rushing through narrow spaces, buck in field, groan when ridden, behind the vertical at trot, catch in field, learning speed, forward going	18		Retained

**Table 3:** Extracted temperament rotated components. SL—strongly loading items (*n*); Alpha—Cronbach Alpha of the number of strongly loaded items; Outcome: Retained—all questions were retained for the final version of the E-BARQ; Excluded—all questions were excluded from the final version; Amalgamated—these questions were combined with similar items, reducing the item number in the final questionnaire version.

E-BARQ-t Components	SL	Alpha	Outcome
1. Strongly avoids items such as bicycles, motor bikes, trucks, horse and cart, umbrellas	12	0.92	Amalgamated
2. Signs of anxiety when alone (trembling, fence walking, vocalising, pawing, sweating, trotting or cantering)	7	0.85	Retained
3. Stands still (e.g., for veterinarian, dentist, feet)	5	0.7	Retained
4. Defensive or aggressive (when led and ridden with other horses in various situations)	6	0.81	Retained
5. Defensive or aggressive (when sponged or hosed)	2	0.59	Excluded
6. Yawns (before, during or after work)	3	0.78	Retained
7. Strongly avoids wild, domestic or uncommon domestic animals (on the ground or when ridden)	6	0.91	Amalgamated
8. Paws or holds foot up during or prior to feeding	3	0.71	Retained
9. Stands still for mounting or walks off after mounting	2	0.74	Retained
10. Pulls back or does not stand still when tied	3	0.8	Retained
11. Defensive or aggressive when groomed	2	0.63	Excluded
12. Stands still (electric clippers on body and face)	2	0.89	Retained
13. Defensive or aggressive when saddled or girthed	2	0.59	Excluded
14. Defensive or aggressive when verbally corrected by owner or other handler	2	0.62	Excluded
15. Strongly avoids plastic bags (on the ground or under saddle) or gets distracted by unfamiliar sights	3	0.76	Retained
16. Strongly avoids children or dogs (on the ground or under saddle)	4	0.82	Retained
17. Chews bark, eats faeces, eats soil	3	0.52	Retained
**Non-loading and Miscellaneous Items**			
Unfamiliar sounds, defensive when signalled to canter, when approached, crib-bites, chews on wood or rugs, dunks hay, opens gates, self-mutilates	19		Retained

## Supplementary Materials

The following are available online at https://www.mdpi.com/2076-2615/10/11/1960/s1, File S1: E-BARQ question items and extracted rotated component, File S2: E-BARQ item loadings.
